# A Depression Recognition and Treatment package for families living with Stroke (DepReT-Stroke): study protocol for a randomised controlled trial

**DOI:** 10.1186/1745-6215-12-105

**Published:** 2011-04-30

**Authors:** Richard J Gray, Phyo K Myint, Frances Elender, Garry Barton, Michael Pfeil, Gill Price, Niki Wyatt, Garth Ravenhill, Ester Thomas, Jenny Jagger, Amelia Hursey, Kelly Waterfield, Sheila Hardy

**Affiliations:** 1 Faculty of Medicine and Health Science, University of East Anglia, Norwich, UK; 2 Medicine for the Elderly, Norfolk and Norwich University Hospitals NHS Trust, Norwich, UK; 3 Clinical Research and Trials Unit, Norfolk and Norwich University Hospitals NHS Trust, Norwich, UK; 4 PhyHWell Project, Northamptonshire Primary Care Teaching NHS Trust, Northampton, UK

## Abstract

**Background:**

Depression occurs in up to 50% of patients after stroke and limits rehabilitation and recovery. Mood disorders are also highly prevalent in carers; their mental health intertwined with the physical and mental wellbeing of the person they are caring for. We argue that working with families, rather than patients alone may improve the treatment of depression in both patients and their carers enhancing the mental wellbeing and quality of life of both.

**Methods:**

A single blind cluster randomised controlled trial to evaluate whether families after stroke who are treated with the Depression Recognition and Treatment package (DepReT-Stroke) in addition to treatment as usual (TAU) show improved mental well being compared to those families who receive only TAU. We aim to recruit one hundred and twenty-six families (63 in each group). The DepReT-Stroke intervention will help families to consider the various treatment options for depression, make choices about which are likely to fit best with their lives and support them in the use of self-help therapies (e.g. computerised Cognitive Behavioural Therapy or exercise). An essential component of the DepReT-Stroke package will be to help people adhere to their chosen treatment(s). The primary outcome will be the Mental Component Subscale of the SF-36 assessed at baseline and again six months post intervention. Effectiveness of the intervention will be determined using analysis of co-variance; comparing the mean change in MCS scores from baseline to six months follow-up adjusting for the clustering effects of baseline scores and family. An economic evaluation of the intervention will help us determine whether the intervention represents a cost-effective use of resources.

**Discussion:**

Depression both for patients and their carers is common after stroke. Our Depression Recognition and Treatment package (DepReT-stroke) may help clinicians be more effective at detecting and managing a common co-morbidity that limits rehabilitation and recovery.

**Trial Registration:**

ISRCTN: ISRCTN32451749

Research Ethics Committee Reference Number: 10/H0310/23

Grant Reference Number: (NIHR) PB-PG-0808-17056

## Background

### Stroke and depression

Stroke is a leading cause of disability, requiring considerable adjustment in the lives of those affected. Depression after stroke (Post Stroke Depression, PSD) is common and, compared with non-depressed stroke patients, is associated with worse long-term outcomes [1-3]. Depression is a distressing illness and is associated with a substantial reduction in quality of life and increased risk of suicide [4-6]. PSD occurs in up to 50% of all stroke survivors [7,8] with prevalence peaking approximately six months after the vascular event^9^. PSD is often inadequately diagnosed and treated, despite an evidence base for effective treatments [9]. Adherence with treatment is often poor, especially when prescribed without taking patient preferences into account. In England, stroke results in an estimated total annual cost of £7 billion [10,11].

### Treatment for depression

There is established efficacy for a range of treatments for depression that include medication, psychological treatments (such as cognitive behavioural therapy (CBT)) and exercise [12]. Antidepressants are effective in PSD both in terms of improving mood and enhancing stroke recovery [8,13]. Problems with the pharmacological treatment of depression seen in the general population are mirrored in patients with stroke. This includes inadequate prescriptions in terms of dose and duration [14,15] and treatment non-adherence: 44% within 3 months of starting treatment [16]. Generally patients express a preference for psychological over pharmacological treatments[17]. Cognitive Behaviour Therapy (CBT) with its problem-focused approach may be particularly suited to the treatment of PSD [18] being as effective as medication with about 50% of patients experiencing clinically meaningful improvement [19]. CBT is the treatment of choice for mild depression and is also recommended for moderate to severe depression or where patients refuse antidepressant treatment. Population studies have shown clear associations between physical exercise and mental health in older adults [20]. Recent trials in patients with PSD have shown exercise to be a feasible intervention that can significantly improve the patient's Activities of Daily Living and perceived physical health as well as help relieve depressive symptoms [20-23].

### The impact of stroke on carers

Many stroke survivors can live in the community, provided they have support and are cared for by a family member [24]. Care giving to a family member who has survived a stroke is demanding [25,26] and is associated with an increased risk of depression in the carer [27]. Patients' poor motor function, impaired memory and behaviour changes seem to have the most profound impact on carers' mental health [26-28]. Three months after the stroke up to 40% of carers will experience a depression, without a noticeable reduction in the prevalence 12 to 18 months after the event [27,29], with some evidence suggesting that care givers were in fact more likely to be depressed than the stroke sufferers themselves [27].

### Study rationale

The mental health of carers is intertwined with the mental health and disease presentation of stroke patients. Literature reviews suggest that stroke rehabilitation should focus on the family rather than the patient alone [30,31]. Stroke survivors whose families act as partners in the rehabilitation process achieve better outcomes [32] and spouses of stroke survivors can benefit significantly in physical and mental well being from nurse-led support and education programmes [33]. Therefore, working with families, rather than patients alone, may have an important role to play in enhancing the mental wellbeing of both group.

While effective management of PSD enhances stroke recovery and improves outcomes [34,35], PSD is often not recognised [35] and consequently treatment is not given. The Healthcare Commission [36] reports that following discharge from hospital, fewer than 30% of stroke patients with emotional problems (such as depression and crying) state they 'get enough help' from the NHS.

We aim to maximise the effectiveness of otherwise established depression treatments in stroke patients and/or carers by enabling them to make informed choices when selecting their depression treatment (i.e. medication, psychological treatments and exercise) and by continuing to support them in structured (adherence) sessions with a trained nurse conducted in their home. The participants' experience of the treatment package will be explored by semi-structured interviews with people with stroke, carers and involved health professionals to understand which aspects of the package are seen as most/least helpful and uncovering any potential barriers to routine use in the health service. This pragmatic approach will yield a much needed evidence based intervention that tests the efficacy mechanism for the prevention, recognition and treatment of depression in families after stroke [37].

The project will enable us to develop a package that will equip nurses (and other health workers) in the community to work effectively and productively in addressing the burden of depression in people with stroke and their carers. Tackling the burden of depression in families after stroke may help ensure optimal disease management leading ultimately to improved health and quality of life for people with stroke and their carers. An economic evaluation of the intervention will help us determine whether the intervention represents a cost-effective use of scarce NHS resources.

## Methods/design

### Trial objectives

To evaluate whether families who receive a Depression Recognition and Treatment package (DepReT-Stroke) in addition to treatment as usual (TAU), show improved mental wellbeing compared to those who receive TAU.

Secondary objectives are to evaluate whether families who receive DepReT-Stroke show improvements in mood, knowledge about depression, adherence to treatment and reduced carer burden compared to families receiving TAU; the process experience of DepReT-Stroke from the point of view of people with stroke, their carers and nurses delivering the intervention; the cost effectiveness of DepReT-Stroke in comparison to TAU.

### Summary of trial design

A single-blind, randomised controlled, cluster trial design to compare DepReT-Stroke with TAU in the treatment of depression in families living with stroke.

### Primary and Secondary Outcome measures

The primary outcome for DepReT-Stroke is change in the mental wellbeing of both patients and carers as measured by the Mental Component Summary (MCS) score of the Medical Outcome Study (MOS) 36 Item Short Form Health Survey (SF-36) version 2.

Secondary outcome measures for both the patient and their primary carer comprise: Hospital Anxiety & Depression Scale (HADS); Knowledge about Depression and Mania Inventory (KDMI); Beliefs about Medicines Questionnaire (BMQ); Zarit Carer Burden Inventory (ZCBI) (only to be completed by the carer); EuroQol quality of life questionnaire (EQ-5D); Levels of resource use associated with DepReT-Stroke and TAU

### Trial Participants

The study population are families living with stroke that have co-occurring depression in the patient, the carer or both. We will recruit 'families' (defined as a person suffering from stroke and their self declared primary carer) from the Norfolk and Norwich University Hospitals (NNUH) NHS Trust Stroke Register [38].

### Inclusion Criteria

The inclusion criteria for DepReT-Stroke are patients diagnosed with ischaemic or haemorrhagic stroke, confirmed by computerised tomography and listed on the NNUH stroke register; three months post stroke; living at home for not less than two weeks; has a self defined 'primary carer'; patient and/or carer scores ≥8 on the HADS; over 18 years of age (no upper age limit).

At three month post stroke the potential complications arising from the acute event should have settled and the patient is more likely to be relatively stable, both medically and psychologically. We chose at least two weeks post discharge from hospital/rehabilitation unit because it can be expected that a sense of routine is beginning to be established by the family by this time. According to the Healthcare Commission's report [36] patients '...can feel abandoned when they lose the emotional and practical support they receive in hospital...' Once patients have settled at home, they, and their families, may welcome further contact with specialist stoke staff.

### Exclusion Criteria

Families living with stroke will not be included in the trial if either the patient or the carer has a serious or unstable medical condition (e.g. advanced/incurable cancer; severe co-morbidity or severe unpredictable pain); psychosis or other severe mental illness; suicidal thoughts or ideation or dementia. We will also exclude families if either the patient or carer is institutionalised (e.g. care home resident) or is participating in any other research concerning stroke or depression

### Screening

The NNUH Stroke Register from 1^st ^January 2005 will be cross referenced with the hospital Patient Administration System (PAS) records to exclude patients who have subsequently died, have evidence of serious/unstable medical conditions or severe mental illness or as far as we can tell are not eligible to be included in the study for any other reason (i.e. discharge destination of residential or nursing home). A further screen for mortality will be conducted by telephoning the patient's GP to request information on their status. The remaining patients and their carers will be written to by the Consultant Stroke Physician who treated them whilst they were in hospital to invite them to participate in the study. The letter contains brief information about the trial, what it involves and includes a check list on the essential inclusion criteria (i.e. that the patient has a self-defined 'primary carer' and that either the patient and/or carer consider they are depressed [39]). If the patient and carer are interested in the study, they will be asked to complete a reply slip and send it back to the patient's consultant in the free post envelope provided. The reply slip requests information on the name of the person who wishes to be contacted by the research team and whether the patient has any difficulties with speaking.

Potential participants/carers will be telephoned by a research nurse who will make further enquiries on trial eligibility and administer the HADS questionnaire to the patient and/or carer as appropriate. If either the patient or carer has a HADS score ≥8 and they still wish to participate in the trial, the nurse will arrange a date for a Research Associate (RA) from the study team to visit them at home. All those approached will be asked whether they wish to receive written information about recognising, preventing and treating depression.

### Informed Consent

A research assistant from the study team will visit the family and make a final check on trial eligibility. Written and verbal versions of a Participant Information Leaflet (PIL) will then be presented to the person with stroke and their carer detailing the exact nature of the study. In particular the RA will explain the randomised allocation element of the trial. It will be made clear that the family is free to withdraw from the study at any time for any reason without prejudice to future care, and with no obligation to give the reason for withdrawal. The patient and carer will be allowed as much time as they wish to consider the information and ask questions. If required, the RA will facilitate opportunities to question other members of the research team, their General Practitioner (GP) or other independent parties to decide whether they will participate in the study. This may mean that the RA has to arrange a subsequent home visit at a later date after prior agreement and arrangement with the patient and the carer.

We will seek consent from the person with stroke whenever possible. An additional PIL has been designed to accommodate people with aphasia and the RA will be trained in effective strategies for communication with this patient group. Written Informed Consent will be obtained from the person with stroke and their carer if they were eligible and agreed to participate. In case of mental incapacity to give informed consent, we will seek approval of an appropriate personal consultee (which we expect will be the carer in most instances). Written information will be provided to potential consultees describing this role under section 32 of the Mental Capacity Act [40] and information about the research similar to that given to participants able to consent for themselves.

### Baseline measures

The RA who is masked to treatment allocation and trained to administer questionnaires in a standardised way will collect baseline data after informed consent is obtained. The following data will be collected:

#### Mental Component Summary of the 36 Item Short Form Health Survey (SF-36v2)

The SF36 is a self-report multidimensional measure of health-related quality of life and wellbeing [41]. The psychometric properties of the SF36 have been well established. The scales of the SF-36v2 address eight health domains and two summary measures are provided: a physical component summary score (PCS), and a mental component summary score (MCS). The MCS was selected as the main quality of life (QoL) outcome measure for this trial as it has been shown to have good sensitivity to change [42]

#### Hospital Anxiety and Depression Scale (HADS)

The HADS is a self screening questionnaire for depression and anxiety [43]. It consists of 14 questions, seven for anxiety and seven for depression. Although it was designed for hospital General Medical Outpatients, it has been extensively used in Primary Care settings [44].

#### Knowledge of Depression Multiple Choice Question Test (KD-MCQ)

The KD-MCQ is a 27-item, multiple choice validated instrument to measure educational domains of knowledge in people suffering from depression [45]. A higher score indicates greater knowledge about depression.

#### Beliefs about Medicines Questionnaire (BMQ)

The BMQ consists of two five items scales assessing peoples' beliefs about the necessity of prescribed medication for controlling their illness and potential consequences of taking it [46]. Participants rate each item on a five point Likert-type scale. A higher score indicates more positive attitudes towards medication.

#### Zarit Carer Burden Inventory - Short version (ZCBI-s)

The ZCBI is a validated instrument used to measure the distress experienced by caregivers of elderly or disabled persons [47]. The questionnaire comprises 22 items about the impact of the person's disabilities on caregiver's physical and emotional health. A truncated version of this instrument (12 items) has been developed which produces results comparable to the full questionnaire [48]. This short version has been selected to reduce the time burden imposed on study participants when administering the questionnaire.

#### The EuroQol EQ-5D questionnaire (EQ-5D)

The EQ-5D, from which Quality Adjusted Life Years (QALYs) can be derived is an established, standardised health-related quality of life instrument used extensively in clinical studies [49]. It comprises five items covering the domains of mobility, self-care, usual activity, pain/discomfort and anxiety/depression. The EQ-5D is cognitively simple, taking only a few minutes to complete. Instructions to respondents are included in the questionnaire.

#### The Client Service Receipt Inventory (CSRI) amended for use in older people

The CSRI [50] collects retrospective information about the interviewee's use of health and social care services, accommodation and living situations, income, employment and benefits. An adapted version of this instrument will be used to collect information on trial participants' use of health services and associated costs incurred over the study period.

### Potential confounding factors

We will measure a number of potential confounding factors at baseline including, stroke severity (using the Stroke Impact Scale [51]), continence (using the Barthel Activities of Daily Living Index - items 5, 6 and 7 [52]), cognitive function (using the General Practitioner Assessment of Cognition [53]), behavioural disturbance (using the Behaviour and Mood Disturbance Questionnaire (BMDQ)[54]), support mechanisms and social contact (using the Measure of Social and Recreational Activities (MSRA))[55], duration of illness: measured in weeks since event, and current medication and any recent change to drug regimes, measured by patient and carer self-reports, self reported co-morbidities and past history of depression.

### Randomisation

All eligible families will be randomly assigned to either the DepReT-Stroke or TAU arm of the trial. Randomisation will be undertaken by the Clinical Research Trials Unit (CRTU) at the University of East Anglia (UEA). Patients will be allocated a unique identification number which will be sent to the trials unit, where allocation will be carried out by permuted blocks of random size. The treating nurse will be notified of the allocation and arrange directly with the patient for the allocated treatment to be given. The RA who conducts the baseline interview and the follow-up assessment will remain blind to allocation throughout the study to minimise bias. The randomisation schedule will be designed by the CRTU.

### Intervention

#### Development of the DepReT-Stroke package

DepReT-Stroke is a comprehensive, evidence-based, structured intervention with associated information in a range of appropriate formats capable of being tailored according to individual need and designed for delivery by a trained nurse specialist. The intervention has been developed by the research team in conjunction with stroke, depression and education experts. Core aims of the package are to facilitate shared decision making between practitioners and people with stroke and/or their carers with regard to the treatment of depression. It will be offered as an adjunct and enhancer of TAU.

DepReT-Stroke will enable families to consider the various treatment options for depression, make choices about which are likely to fit best with their lives and support them in the use of self-help therapies (e.g. computerised CBT or exercise). An essential component of the DepReT-Stroke package will be to help people adhere to their chosen treatment(s) by enabling them to explore possible ambivalence towards and beliefs about antidepressive treatments (both psychological and pharmacological). Sessions will focus on exchange of information about therapy choices and facilitate reflection on progress to date. The package will also be designed to help the person in the family who may not be depressed to adjust to life after a stroke, consider the possible risk of depression in the future and provide them with techniques to self monitor their mood. As aphasia is an important predictor of carer strain, particular care will be taken to design the DepReT-Stroke package in a manner that is relevant for aphasic patients.

#### Description of the DepReT-Stroke package

The intervention will comprise six sessions with a trained study nurse delivered at fortnightly intervals. Each session will last approximately 30 minutes. In addition, two booster sessions will be delivered at three and five months post randomisation. These sessions will enable the nurse to monitor adherence, facilitate participants to reflect on progress and help them to adapt or refine their chosen treatment strategies if required. There will be a strong emphasis on facilitating homework tasks and self help activities. Risk of suicide will be assessed at the beginning of each session and, if identified, the participant's GP or the local crisis team will be contacted. Completion of treatment will be defined as attending a minimum of five out of eight sessions with the nurse (including booster sessions). After the first 20 sessions the nurses who deliver the package will feed back their experiences of delivering the DepReT-Stroke package to the research team. If any gaps in information or difficulties with format are identified, these will be addressed before the remaining sessions are delivered.

### Follow up

Baseline measures of efficacy will be repeated at six months post randomisation; reflecting the recommended duration of treatment for moderate to severe depression^2^. The RA blinded for trial arm allocation will telephone families to arrange a date to visit them at home to obtaining follow up information.

### Analysis

#### Description of Statistical Methods

Standard methods will be used to provide tabular and graphical summaries as appropriate for continuous and categorical variables.

#### Baseline analyses

To determine external generalisability, demographic and clinical characteristics of participants' responses at the baseline phase of the study will be compared for participants who are subsequently randomised and participants who are screened but not randomised using one-way analysis of variance or Mann-Whitney test for continuous variables and Fishers exact or Chi-squared test for categorical variables, as appropriate. The specific criteria by which participants are excluded from randomisation will be tabulated. Demographic and clinical characteristics will be compared between the intervention and control groups to identify any imbalances without statistical testing [56].

#### Sample size

The cluster within this trial is the family (patient and carer). We will need to analyse data from 102 individuals (i.e. 51 families) (approximately 51 individuals in the DepReT-Stroke group and 51 individuals in the TAU group). This will provide 80% power to detect, with a significance level 5%, an overall difference between intervention and TAU of 6 points in the SF-36v2 MCS sub-scale with a standard deviation of the MCS of 12 [57] equivalent to an effect size of 0.5SD. The calculation assumes a clustering effect of scores by family with a ICC of 0.05, and that the analysis would adjust for baseline values, with a baseline-endpoint correlation in MCS scores of 0.5. With an estimated 20% attrition rate, this will require randomising 126 individuals at baseline. We pre-defined 0.5 SD (6 points) difference between intervention and TAU as this effect size is clinically significant difference for stroke patients/carers with depression [58].

#### Effectiveness analysis

The effectiveness of the intervention on the primary outcome (MCS-SF-36v2) will be assessed by comparing the mean change in values of DepReT-Stroke and TAU groups from baseline to six month follow-up, adjusting for the clustering effect of the family and the baseline score. This will be achieved by analysis of covariance with robust standard errors using the STATA statistical software package (STATACORP LP, Texas, USA). The effects of the intervention will be reported as change scores. Appropriate adjustments will be made in the statistical analyses for prognostic factors, particularly those unevenly distributed between the groups. Prognostic strength will be checked with regression analyses and only those with strongest effects on outcome used to adjust the trial effects [59,60].

Both intention to treat (ITT) and per protocol (PP) analyses will be performed. ITT analysis will be the primary analysis population and will be used for evaluation of all endpoints.

### Economic evaluation

In line with guidance from the National Institute for Health and Clinical Excellence (NICE), costs will be calculated from the perspective of the NHS and personal social services and encompass those costs that are potentially related to the intervention in question. We will monitor the levels of resource use associated with any re-admission to hospital and other health and non-health care contacts (e.g. further therapy, nursing care, social services). Patient and carer resource use will be obtained from responses to a modified version of the Client Service Receipt Inventory (CSRI) at baseline and six months post randomisation. Appropriate unit costs will subsequently be assigned in order to calculate total costs for DepReT-Stroke and TAU.

The measures of effectiveness employed in the economic analysis will be the EQ-5D [49]. This is a generic measure of health status designed to compare the benefits of different interventions. It has 5 dimensions - mobility, self-care, usual activities, pain, anxiety and depression. These will be used to calculate quality-adjusted-life-years (QALYs) associated with the intervention and TAU.

An economic model will be constructed in order to estimate both the mean overall cost and effect associated with both DepReT-Stroke and treatment as usual. If one of these options were shown to be less costly and more effective than the other then this would suggest that it 'dominates' the other, and represents a cost-effective use of scarce resources. Alternatively, the incremental cost-effectiveness ratio associated with DepReT-Stroke will be estimated and assessed in relation to a range of cost-effectiveness thresholds per QALY. The associated level of uncertainty will also be characterised by estimating the cost-effectiveness acceptability curve. Sensitivity analysis will also be undertaken to assess the robustness of conclusions to changes in key assumptions.

### Qualitative evaluation

Qualitative interviews will be undertaken with a purposively selected sub-sample of patients (n = 5) and carers (n = 5) from the intervention group. A 'short list' of suitable participants will be compiled as follows. At the follow up visit the researcher will enquire whether families are still willing to undertake this element of the study. If so, they will be placed into one of three groups (i.e. the person with stroke is depressed, the carer is depressed or both are depressed). Every second family in each group will be selected and the researcher will arrange a convenient time for another member of the research team to visit. Interviews will be conducted in families' homes in accordance with a defined schedule and take approximately 30 minutes to complete. The aim of the interviews will be to: obtain insights into families and health professionals' experiences of using the package; consider which elements of the package were perceived as being most and least helpful; explore the participants' perceptions of the effect that they think the package (as opposed to just the antidepressant treatment) has on them; Uncover any potential barriers and roadblocks to using the package; explore how the package could be refined and enhanced.

All interviews will be audio-recorded, transcribed verbatim and transcripts sent to participants for corroboration. Confirmed transcripts will be coded using thematic analysis [61]. A summary of the results will then be returned to participants for member checking [62]. If the intended sample (n = 10) fails to provide a rounded picture of delivering DepReT-Stroke, additional participants will be interviewed using the same methodology as described until saturation is achieved. At the end of the intervention phase, a similar interview will be conducted with the nurse delivering DepReT-Stroke. This will take place at the University of East Anglia at a mutually convenient time.

### Potential Bias

In order to compensate for any possible "researcher bias" the following elements have been included in the research design. A single nurse will deliver all DepReT-Stoke sessions. The subjective nature of the self report instruments used for evaluation of the intervention is accepted and every effort will be made to minimise potential bias due to this dynamic. In particular, people with stroke and their carers may over or under report health status depending on the trial arm to which they have been assigned. The researcher who conducts the baseline interview and the follow-up assessment will be trained to administer questionnaires in a standardised way. Every effort will be made to ensure they remain blind to trial arm allocation throughout the study. At the follow up meeting they will request that the family do not mention whether they have seen the nurse or not. However, economic evaluation data on use of health services is likely to reveal trial arm allocation. For this reason the researcher will obtain this information after all other questionnaires have been completed. Information regarding potential confounding factors (listed above) will be collected at baseline. Qualitative interviews will not be conducted with the first 5 families in each sub-group (i.e. the person with stroke is depressed, the carer is depressed or both are depressed) in order to allow the intervention to settle.

### Ethical considerations

The trial protocol was reviewed by a NHS Research Ethics Committee (REC) and was approved from an ethical perspective (REC reference number: 10/H0310/23).

## Discussion

Depression is a distressing and disabling illness and commonly occurs after stroke in both patients and their carers. Our DepReT-Stroke package aims to ensure that families develop a better understanding of depression and are able to choose a treatment that suits them and they are comfortable sticking with. DepReT-Stroke is a manualised intervention with the express intention that if effective health professionals will be able to incorporate it into their practice with minimal training. We need, however, to establish the efficacy and cost effectiveness of the package using the most rigorous scientific approach.

## Abbreviations

BMDQ: Behaviour and Mood Disturbance Questionnaire; BMQ: Beliefs about Medicines Questionnaire; CRTU: Clinical Research Trial Unit; CSRI: Client Service Receipt Inventory; DepRET: Depression Recognition and Treatment; EQ-5D: EuroQol quality of life questionnaire; GP: General Practitioner; HADS: Hospital Anxiety & Depression Scale; ITT: Intention to Treat; KDMI: Knowledge about Depression and Mania Inventory; MCS: Mental Component Sub-scale; MSRA: Measure of Social and Recreational Activities; NHS: National Health Service; NICE: National Institute for Health and Clinical Excellence; NIHR: National Institute for Health Research; NNUH: Norfolk and Norwich University Hospitals; PAS: Patients Administration System (PAS); PP: Per Protocol; PIL: Participant Information Leaflet; PSD: Post Stroke Depression; QALYS: Quality Adjusted Life Years; RA: Research Associate; REC: Research Ethics Committee; SF-36: Short Form-36; TAU: Treatment As Usual; UEA: University of East Anglia; ZCBI: Zarit Carer Burden Inventory;

## Competing interests

Richard Gray has provided consultancy work to AstraZeneca, Bristol-Myers Squibb, Jannsen Cilag, Eli Lilly and Co. Otsuka Pharmceutical Europe Ltd, Pfizer, received honoraria from AstraZeneca, Bristol-Myers Squibb, Jannsen Cilag, Eli Lilly and Co. Otsuka Pharmceutical Europe Ltd, Pfizer, Wyeth and had research support from AstraZeneca.

## Authors' contributions

RJG, PKM, FE, GB, MP, PD and GP contributed to the design of the study. All authors contributed to the development of the trial protocol and data collection. RJG, PKM and FE drafted the paper. All authors provided input into revisions of the paper and have approved the final manuscript.
